# Biobehavioral phenotypes of chronic low back pain: Psychosocial subgroup identification using latent profile analysis

**DOI:** 10.1093/pm/pnaf095

**Published:** 2025-07-25

**Authors:** Fatemeh Gholi Zadeh Kharrat, Prakruthi Amar Kumar, Wolf Mehling, Irina Strigo, Jeffrey Lotz, Thomas A Peterson, REACH Investigators, Jamie Ahn, Kristina Benirschke, Alexandra Bryson, Katherine Bunda, Briana Davis, Carolina Dorofeyev, Rosalee Espiritu, Pirooz Fereydouni, Aamna Haq, Nicholas Harris, Sara Honardoost, Gabriel Johnson, Jennifer Johnson, Edward Lingayo, Robert Miller, Phirum Nguyen, Christopher Orozco, Lindsay Ruiz-Graham, Kie Shidara, Kaitlyn Smith, John (Boyuan) Xiao, Michelle Yang

**Affiliations:** Department of Orthopaedic Surgery, University of California, San Francisco, CA, United States; Bakar Computational Health Sciences Institute, University of California, San Francisco, CA, United States; Department of Neurological Surgery, University of California, San Francisco, CA, United States; Department of Family and Community Medicine, Osher Center for Integrative Health, University of California, San Francisco, CA, United States; Emotion and Pain Laboratory, San Francisco Veterans Affairs Health Care Center, San Francisco, CA, United States; Department of Psychiatry, University of California, San Francisco, CA, United States; VA Advanced Imaging Research Center, San Francisco Veterans Affairs Health Care Center, San Francisco, CA, United States; Department of Orthopaedic Surgery, University of California, San Francisco, CA, United States; Department of Orthopaedic Surgery, University of California, San Francisco, CA, United States; Bakar Computational Health Sciences Institute, University of California, San Francisco, CA, United States

**Keywords:** chronic low back pain (cLBP), biobehavioral, latent class modeling (LCM), latent profile analysis (LPA)

## Abstract

**Objective:**

This study identifies distinct biobehavioral phenotypes among patients with chronic low back pain (cLBP) using Latent Profile Analysis (LPA).

**Methods:**

These phenotypes were derived from baseline data from two cohorts within the NIH HEAL BACPAC consortium: BACKHOME, a large nationwide e-cohort (*N* = 3025) utilized for model training, and COMEBACK as external test set, a deep phenotyping cohort (*N* = 450) utilized for generalization. The analysis incorporated variables including pain characteristics, psychosocial factors, lifestyle habits, and social determinants of health. Model fit was optimized via 10-fold cross-validation with 100 bootstraps and evaluated using Akaike Information Criterion (AIC), Bayesian Information Criterion (BIC), and Entropy(uncertainty).

**Results:**

Four classes were identified: Class 1 (“High Distress and Maladaptive Behaviors”) displayed high levels of anxiety, depression, and fear avoidance. Class 2 (“Resilient and Adaptive Coping”) exhibited low maladaptive behaviors and high pain self-efficacy. Class 3 (“Intermediate Maladaptive Patterns”) represented moderate levels of psychological and behavioral challenges, while Class 4 (“Emotionally Regulated with High Pain Burden”) demonstrated strong emotional regulation despite significant pain burden. Class sizes were 701, 413, 893, and 947 for the train set, and 127, 108, 95, and 68 for the test set, respectively. Fit metrics supported the model’s performance and generalizability (BACKHOME (train set): AIC = 77 792, BIC = 78 338, Entropy = 0.82; COMEBACK(test set): AIC = 72 437, BIC = 73 880, Entropy = 0.81). Statistical analysis revealed significant differences between classes (*P* < .05) in key variables such as pain self-efficacy, fear avoidance, and emotional awareness, and changes in pain severity and health-related quality of life over time (*P* ≤ .001), indicating clinical utility.

**Conclusions:**

Our findings highlight the heterogeneity of cLBP and suggest that tailored treatments targeting these distinct subgroups could improve clinical outcomes. This work advances our understanding of cLBP by providing a robust framework for identifying patient subgroups based on biobehavioral characteristics. Results underscore the value of LPA in uncovering clinically meaningful patterns in complex conditions like cLBP, paving the way for more personalized treatment approaches.

## Teaser

This study used Latent Profile Analysis (LPA) to identify four biobehavioral subgroups of individuals with chronic low back pain (cLBP) across two independent cohorts. Four classes were identified:Class 1 (“High Distress and Maladaptive Behaviors”), Class 2 (“Resilient and Adaptive Coping”), Class 3 (“Intermediate Maladaptive Patterns”), and Class 4 (“Emotionally Regulated with High Pain Burden”). These findings highlight the utility of LPA in revealing clinically meaningful phenotypes in cLBP, supporting the development of more personalized and targeted treatment strategies.

## Introduction

One of the most prevalent illnesses in the general population is low back pain (LBP) with a lifetime prevalence over 80%. It is a leading cause of disability, medical expense,^1^^,^^2^ and activity limitation in the United States for individuals under 45, the second most common reason for family doctor visits, the third most common reason for surgical operations, and the fifth most common reason for hospital admissions,.^3–5^ Research has highlighted the importance of biobehavioral factors in the development and persistence of cLBP, including personality, environmental stressors, cognitive processes, and physiological correlates.^6^^,^^7^ The interplay of biological, environmental, and psychological processes contributes to pain and disability in chronic low back pain (cLBP) patients.^7^ Epidemiological research suggests that the development of chronic pain involves dynamic interactions between physiological, psychological, and social factors, leading to variability in pain experiences over time.^8^ A fear avoidance model demonstrates the cognitive, emotional, behavioral, biomedical, and social factors involved in cLBP.^9^^,^^10^ Cognitive-behavioral therapy and other psychological interventions have demonstrated efficacy in managing cLBP and are part of clinical guidelines.^11^ Given the complexity and variability of chronic pain, a comprehensive assessment of these psychosocial factors is essential for effective classification, understanding, and customization of cLBP management to individual patients.^12^^,^^13^

Latent class analysis (LCA) and latent profile analysis (LPA), a type of LCA for continuous variables, are person-centered techniques that determine latent subpopulations within a population based on multiple measured variables.^14^^,^^15^ LPA, commonly attributed to Lazarsfeld and Henry,^16^ is a type of mixture modeling that classifies individuals into homogenous subgroups, known as latent profiles, based on their response patterns to observed variables. Unlike traditional statistical techniques, which might miss these latent structures and interactions, LPA can model complex, non-linear relationships between variables, particularly helpful in healthcare. It can capture complex relationships among variables to provide more accurate phenotyping.^17^^,^^18^ Recent studies have used LPA to determine distinct subgroups among chronic pain patients, indicating the complexity of pain experiences and treatment needs. For example, Walton et al.^19^ applied LPA to Canadian military veterans. They identified five distinct profiles, displaying the heterogeneity of chronic pain experiences and underscoring the need for individualized treatment strategies. Carlesso et al.^20^ used LPA among workers with chronic low back pain (cLBP) to outline pain profiles based on intensity, quality, and psychosocial characteristics. Their findings highlighted the relevance of protective factors such as pain self-efficacy and social support, which can inform the development of more targeted and holistic care pathways for individuals with high pain burdens. Obbarius et al.^21^ found four subgroups based on pain burden and emotional distress in patients undergoing multimodal inpatient treatment. Rovner et al.^22^ determined four patient clusters based on pain acceptance levels, which were associated with specific patterns of psychological and physical function. SY Kim et al.^23^ conducted LPA. They used data from the National Survey of Midlife Development in the United States (MIDUS) to study heterogeneity in chronic pain, depression, anxiety, and somatic amplification (PDAS) symptoms. They identified three patient clusters that primarily differed in symptom severity rather than type. These results highlight the clinical utility of person-centered approaches in understanding PDAS comorbidities and informing targeted intervention strategies. According to these results, latent class modeling may help capture chronic pain’s complex, multidimensional nature. By defining clinically meaningful subgroups based on behavioral and psychosocial patterns, this person-centered method facilitates the development of more personalized and adequate interventions tailored to individual patient profiles.

In this study, we hypothesized that multiple distinct latent classes exist within our patient population, each defined by unique biobehavioral patterns. The analyses were conducted using baseline data from the Chronic Low-Back Pain in Adults Study (BACKHOME) as train set. To confirm the stability of class size and characterization, baseline data of the Clinical Cohort for Comprehensive Deep Phenotyping of Chronic Low-Back Pain in Adults Study (COMEBACK), which served as the external test set. Both datasets are part of the BACPAC consortium for the NIH HEAL initiative.^24^^,^^25^ Our objective was to determine the presence of latent classes with unique biobehavioral characteristics.

## Material and methods

### Data source

The University of California, San Francisco (UCSF) Core Center for Patient-centric, Mechanistic Phenotyping in Chronic Low Back Pain (REACH) is 1 of the 3 NIH Back Pain Consortium (BACPAC) Research Programs Mechanistic Research Centers (MRCs). REACH^25^ is conducting two large investigator-initiated translational research cohort studies called: The BACKHOME and the COMEBACK studies.^25^  **Train set**: The BACKHOME study is a site-less longitudinal observational e-cohort of 3025 individuals. To our knowledge, BACKHOME is the largest prospective remote registry of nationwide adults with cLBP. **Test set:** The COMEBACK is a longitudinal multicenter in-person observational study of 450 adults with chronic low back pain designed to perform comprehensive deep phenotyping.

### Variable selection

The data we use include variables for various categories (for detailed insights on the meanings of acronyms, refer to (Appendix SA): demographic information such as age, sex; anthropometrics, such as Body Mass Index (BMI); pain-related measures such as the PEG score and PROMIS Pain Interference; pain characteristics including duration, frequency, and intensity; quality of life assessed through the PROMIS Physical Functioning (SF 6b) Score; mood, such as depression and the Pain Anxiety Symptoms (PAS); the most common parameters for pain persistence, such as fear avoidance by Fear Avoidance Beliefs with Physical Activity (FABQ-P), pain catastrophizing by Pain Catastrophizing Scale SF (PCS-6), and pain self-efficacy by Pain Self-Efficacy Questionnaire (PSEQ-4); and lastly elements of interoceptive awareness, such as Non-Distracting (the tendency not to ignore or distract oneself from sensations of pain or discomfort), Emotional Awareness (one’s awareness of the connection between body sensations and emotional states), Self-Regulation (one’s ability to regulate distress by attention to body sensations) from the Multi-dimensional Assessment of Interoceptive Awareness version 2 questionnaire (MAIA-2).

The selected variables encompass a range of biobehavioral, psychological, and pain-related measures, providing a robust foundation for the latent profile analysis (LPA) model. These variables were chosen for their established relevance in cLBP research and their ability to capture critical dimensions of the condition, such as pain intensity, psychosocial impact, emotional regulation, and interoceptive awareness, and including diverse measures ensured that the LPA model effectively identified meaningful and clinically interpretable latent classes.^26^ This selection ensured that the analysis aligned with the study’s primary objective of uncovering actionable patient phenotypes.

### Statistical methods

LPA(16) was used to discover groupings of patients with identical unique biobehavioral patterns within our study population. In more technical terms, LPA is used to detect latent (or unobserved) heterogeneity in samples. To achieve a parsimonious model, we aimed to select the fewest number of latent classes that still sufficiently described the population. From a substantive perspective, the model and chosen class count were expected to align with theoretical and practical considerations such as clinical interpretability, existing literature on cLBP, and the distribution of key biobehavioral measures.^26–28^

We used the StepMix package v2.2.1 in Python for the LPA model development and analysis. Model development and evaluation were accomplished in two phases.

### Phase 1: Model building

We trained the LPA model using the BACKHOME dataset. To determine the optimal number of classes, we employed 10-fold cross-validation with 100 bootstraps and evaluated model fit using Akaike Information Criterion (AIC), Bayesian Information Criterion (BIC) and entropy (uncertainty) to optimize model fit. AIC and BIC were used to balance fit and complexity, while entropy (uncertainty) measured the separation of latent classes.

### Phase 2: Model evaluation

To examine differences between classes, we conducted a 1-way ANOVA with Bonferroni correction on key variables. The Bonferroni correction was applied to account for multiple comparisons, lowering the risk of false positives by adjusting the significance threshold using the formula α_adjusted = 0.05/number of comparisons.^29^ Statistically significant differences across classes were indicated by a *P* value less than 0.05 and verifying the robustness of the class distinctions. Finally, to evaluate the model’s generalizability, we did not re-run the full range of classes (1 to 10) in the COMEBACK. Instead, we applied the optimal number of latent classes, determined from the much larger BACKHOME dataset to the COMEBACK dataset, assessing fit criteria (AIC, BIC, and entropy) and comparing key variable patterns across datasets.

## Results

### Sample characteristics

Tables 1 provides an overview of the demographic characteristics in the study population for the train and test sets, respectively. In the train set, participants were predominantly female (68.1%) and White (85.8%), with 92.3% identifying as non-Hispanic or Latino. In the test set, participants were also largely female (57.1%) and White (75.7%). The test set participants were slightly younger compared to the train set. The train set had a slightly higher mean BMI compared to the test set.

**Table 1.** Sociodemographic characteristics.

Variables	BACKHOME (Train set) (*N* = 3025)	COMEBACK (Test set) (*N* = 450)
**Age**		
Mean (SD)	56.5 (14.2)	55.6 (15.6)
Median [Min, Max]	58 [20,94]	58.5 [18,91]
Missing*	5 (0.16 %)	
**Gender**		
Female	2013 (68.1 %)	257 (57.1 %)
Male	941 (31.8 %)	193 (42.8 %)
**BMI**		
Mean (SD)	29.91 (7.5)	26.76 (5.3)
Median [Min, Max]	28.46 [13.3,71.3]	26.25 [7.8,43.9]
Missing*	13 (0.4 %)	12 (2.6 %)
**Education**		
Did not complete high school	6 (0.2 %)	3 (0.6 %)
Some secondary school (or high school) education	47 (1.5 %)	4 (0.8 %)
High school complete	529 (17.4 %)	46 (10.2 %)
Associate’s or Technical degree complete	525 (17.3 %)	46 (10.2 %)
College or baccalaureate degree complete	948 (31.3 %)	190 (42.2 %)
Doctoral or postgraduate education	970 (32.0 %)	161 (35.7 %)
**Race**		
White	2596 (85.8 %)	341 (75.7 %)
Asian	87 (2.8 %)	45 (10.0 %)
Black or African American	142 (4.6 %)	28 (6.2 %)
Native Hawaiian or Pacific Islander	6 (0.20 %)	4 (0.8 %)
American Indian/Alaska Native	20 (0.6 %)	2 (0.4 %)
More than 1 race	112 (3.7 %)	12 (2.6 %)
Unknown or not reported	62 (2.05 %)	18 (4.0 %)
**Ethnicity**		
Not Hispanic or Latino	2793 (92.3 %)	392 (87.1 %)
Hispanic or Latino	185 (6.1 %)	51 (11.3 %)
Prefer not to answer or Unknown	47 (1.5 %)	7 (1.5 %)

a Observation with missing values were excluded from the analysis.

### LPA model development

**Figure 1.**
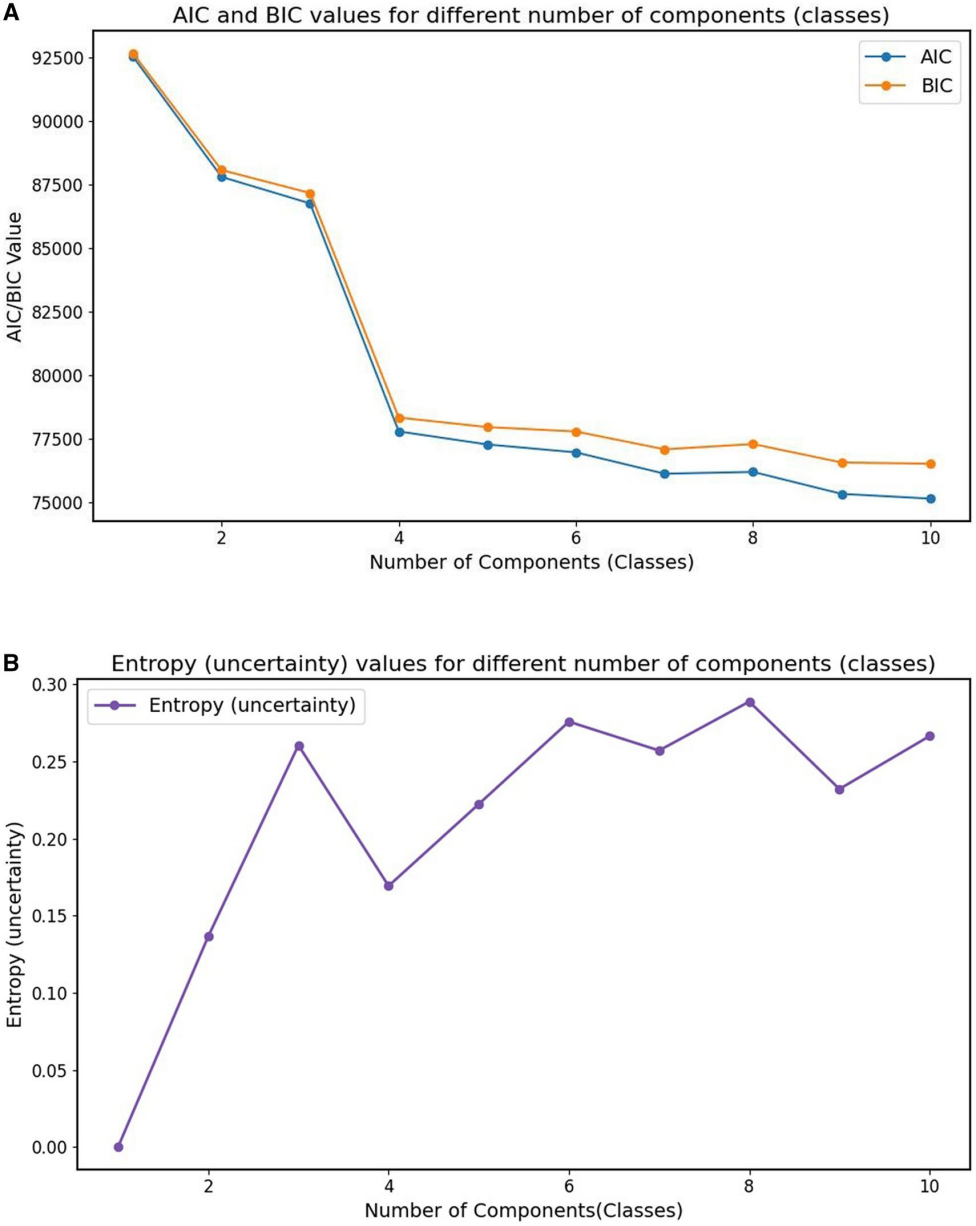
Model selection based on AIC, BIC, and entropy. Abbreviations: AIC, Akaike Information Criterion; BIC, Bayesian Information Criterion. (A) AIC and BIC. (B) Entropy (Uncertainty)

Figure 1 displays the evaluation metrics used to determine the optimal number of components (classes) (range 0–10) for our latent class model. The model fit is revealed as complexity rises in the first graph, which shows AIC and BIC values over a range of class numbers. With each extra class, both AIC and BIC show a steep initial fall, suggesting better model fit; but, after 4 classes, they plateau (Figure 1A). According to this pattern, the explanatory power of the model is not significantly improved by adding more classes after this point in relation to the complexity increase. The second graph (Figure 1B) illustrates the distinct and well-separated classes indicated by displaying the entropy values for each class.

According to the AIC, BIC, and entropy results, we chose models with a better fit with the four classes that provide a strong framework that effectively and efficiently identifies latent structure in the data by capturing the underlying patterns without becoming overly complicated. Consequently, the best-fitting model consisted of four classes. This choice was consistent with best standards for modeling, which balance interpretability and statistical fit to prevent overfitting and preserve significant, interpretable outcomes.

### LPA-Derived class profiles and fit indices

The model was developed using the BACKHOME (train set) and the COMEBACK (test set) dataset with four latent classes. Generalization was evaluated based on class size, fit metrics, and consistency of class patterns across datasets. The class sizes for the train and test sets are shown in Table 2. The model’s robustness is supported by the similar class sizes between the two data sets, which point to a consistent distribution of class membership across datasets. Additionally, measures including the AIC, BIC, and entropy were used to assess the model fit. For the BACKHOME, train set, the AIC was 77 792, BIC was 78 338, and entropy was 0.82. In the COMEBACK, test set, the AIC was slightly lower at 72 437, with a BIC of 73 880 and an entropy of 0.81. The model’s structural integrity is preserved across datasets, indicating strong external validity, as evidenced by the similarity in fit metrics between the train and test sets.

**Table 2.** Class size.

Data set	Class 1	Class 2	Class 3	Class 4
Train set (BACKHOME)	701	413	893	947
Test set (COMEBACK)	127	108	95	68

### Biobehavioral characterization of latent classes

**Figure 2.**
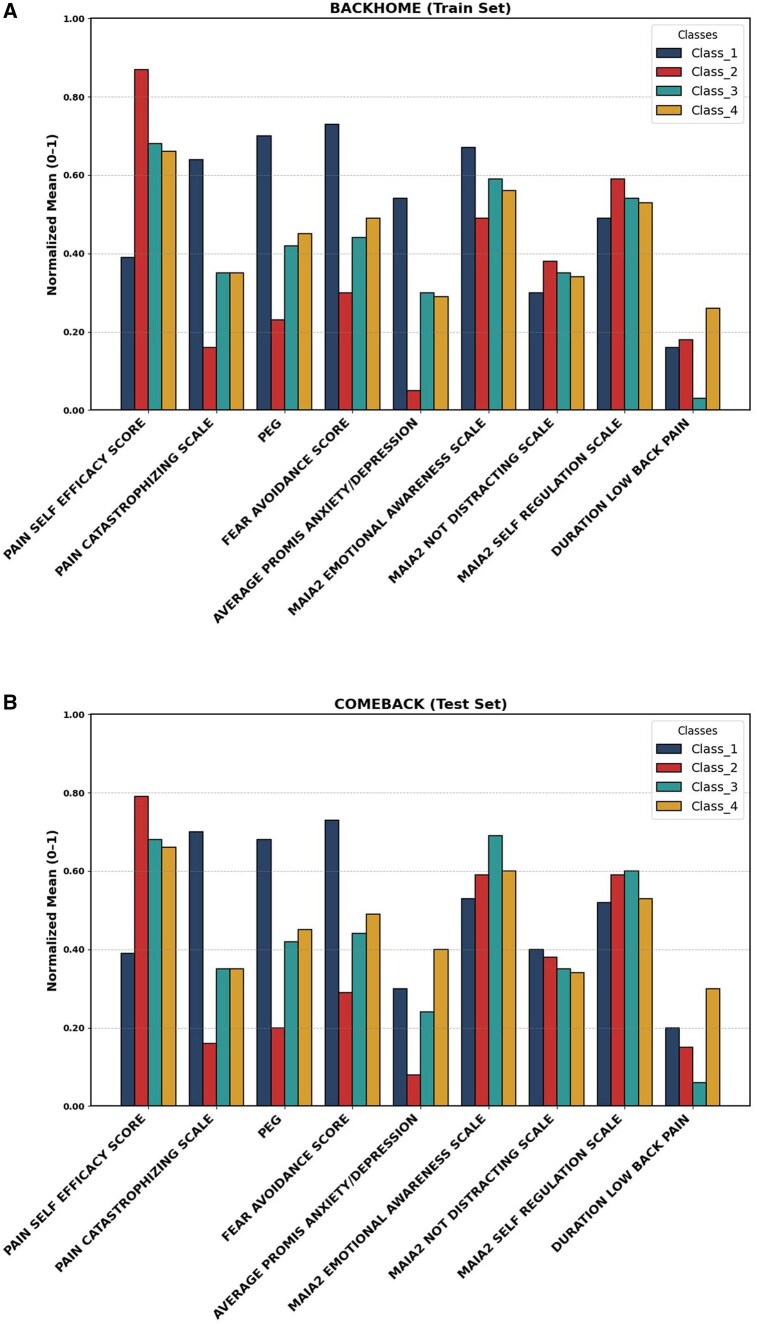
Comparisons of normalized class means in (A) Train set and (B) Test set. (A) Train set. (B) Test set.

The class specific profiles of normalized indicator mean for four latent classes in the BACKHOME (train set), and COMEBACK (test set) are compared in Figure 2 using measures like the PEG, FABQ, PSEQ, PCS6, PROMIS Anxiety/Depression, MAIA-2 Emotional Awareness, MAIA-2 Not Distracting, and MAIA-2 Self-Regulation. To facilitate comparability across variables with different units, the class specific means of the standardized indicators were rescaled to a 0–1 range after model estimation. These values reflect the relative levels of each indicator across latent profiles.

Each class in the BACKHOME (train set) has distinctive mean profiles highlighting qualities exclusive to each scale. The 4 latent classes identified in the model were labeled to reflect their dominant characteristics: Class 1 (“High Distress and Maladaptive Behaviors”), Class 2 (“Resilient and Adaptive Coping”), Class 3 (“Intermediate Maladaptive Patterns”), and Class 4 (“Emotionally Regulated with High Pain Burden”). Class 1 members displayed the highest means of anxiety/depression, fear avoidance, PCS6 and PEG, indicating a higher likelihood of displaying higher pain and avoidance behaviors. Class 2 exhibits comparatively low means for maladaptive scores on these measures, which might be indicative of a profile with higher pain self-efficacy and less challenges with pain management. The included MAIA scales were not markedly different between classes, and although differences exist between classes 1 and 2, they contribute much less to the latent class separation. Other classes, such as Class 3, show intermediate means on several scales, reflecting a range of psychological patterns in the population. Class 4 shows higher pain-related scores (eg, PEG and fear avoidance) but also displayed relative emotional awareness and self-regulation strengths. These capture the distinct behavioral phenotypes and provide a framework for interpreting the latent structure in the data.

The model’s resilience and generalizability are determined by the COMEBACK, which largely reproduces the patterns seen in the train set. The 4-class structure is stable, as evidenced by the comparable patterns in the variable scores between the two sets and the constant high likelihood for Class 1 in maladaptive pain scores, depression/anxiety, fear-avoidance and catastrophizing. For example, Class 1 demonstrated marginally higher mean scores on several MAIA-2 subscales in the test set and significantly lower means for PEG and anxiety/depression, although maintaining its characterization as the most distressed subgroup. Smaller discrepancies in the mean profiles between the train and test sets, may indicate nuanced differences in class composition, reflecting the natural variability within new samples. This comparison between the train and test sets shows that the 4-class model not only finds important latent structures but also maintains stability and interpretability when applied to new data.

### Variable differences across latent classes

After identifying the class membership for the selected variables, we conducted a one-way ANOVA test to assess significant differences across the four latent classes for each variable. Bonferroni corrections were applied to control for multiple comparisons, with a significance level of (*P* < .05). The results are indicated in Appendix SB, indicating *P* values for each variable in both the train (BACKHOME) and test (COMEBACK) data sets; Z scores are shown in Appendix SB. All the variables in the train set, including MAIA2 Emotional Awareness, MAIA2 Not Distracting, MAIA2 Self-Regulation, Duration of Low Back Pain, PEG, PCS6, Pain Self-Efficacy, Anxiety and Depression, and FABQ showed significant differences across the 4 classes, with *P* values of less than .05. The variables in both data sets show significant variations among the 4 classes, according to the ANOVA results. This consistency between the train and test sets highlights different behavioral and psychological characteristics within the latent structure and supports the 4-class model’s stability and interpretability. These results imply that the variables chosen successfully distinguish the latent classes, offering insightful information for further investigation and interpretation.

### Demographic differences across latent classes

Demographic characteristics of the four latent classes are demonstrated in Table 3. Class 1 was consistently younger and had a higher BMI group across both data sets, with a mean age of approximately 51 years and the highest BMI. This class also had more females, particularly in the train set (76.0%). Class 2 was the oldest and most highly educated group, with mean ages over 62 years and the lowest BMI in both data sets. Classes 3 and 4 showed intermediate demographics with modest variation, and demographic distinctions between them were less noticeable. Across all classes, most participants identified as not Hispanic or Latino, with proportions ranging from 85.8% to 94.9%, reflecting overall consistency in ethnic composition.

**Table 3.** Demographic differences across latent classes the (A) Train set and (B) Test set.

A:				
Variable	Class 1	Class 2	Class 3	Class 4
	(*n* = 701)	(*n* = 413)	(*n* = 893)	(*n* = 947)
Age	50.99 (12.67)	62.47 (12.95)	55.77 (15.00)	58.86 (13.76)
BMI	33.19 (9.30)	26.40 (4.45)	29.36 (6.92)	29.53 (6.61)
Gender				
Female	533 (76.0%)	236 (57.1%)	610 (68.3%)	636 (67.1%)
Education				
Less than High School	23 (3.3%)	6 (1.5%)	9 (1.0%)	11 (1.2%)
High School or Technical Degree	398 (56.8%)	70 (17.0%)	256 (28.7%)	292 (30.8%)
Bachelor’s Degree	183 (26.1%)	132 (32.0%)	300 (33.6%)	320 (33.8%)
Doctoral/Postgraduate Degree	97 (13.8%)	205 (49.6%)	328 (36.7%)	324 (34.2%)
Ethnicity				
Not Hispanic or Latino	625 (89.2%)	392 (94.9%)	823 (92.2%)	893 (94.3%)

B:				
Variable	Class 1	Class 2	Class 3	Class 4
	(*n* = 127)	(*n* = 108)	(*n* = 68)	(*n* = 95)
Age	51.70(14.45)	62.78 (15.16)	51.65 (17.28)	55.97 (14.39)
BMI	28.63 (5.82)	25.58 (3.97)	26.21 (6.29)	28.35 (6.03)
**Gender**				
Female	68 (53.5%)	63 (58.3%)	40 (58.8%)	57 (60.0%)
**Education**				
Less than high school	1 (0.8%)	1 (0.9%)	0 (0.0%)	2 (2.1%)
High school or technical degree	28 (22.0%)	9 (8.3%)	16 (23.5%)	31 (32.6%)
Bachelor’s degree	47 (37.0%)	45 (41.7%)	32 (47.1%)	42 (44.2%)
Doctoral/Postgraduate degree	51 (40.2%)	53 (49.1%)	20 (29.4%)	20 (21.1%)
**Ethnicity**				
Not Hispanic or Latino	109 (85.8%)	93 (86.1%)	62 (91.2%)	83 (87.4%)

### Association of latent classes with changes in clinical outcomes

**Figure 3.**
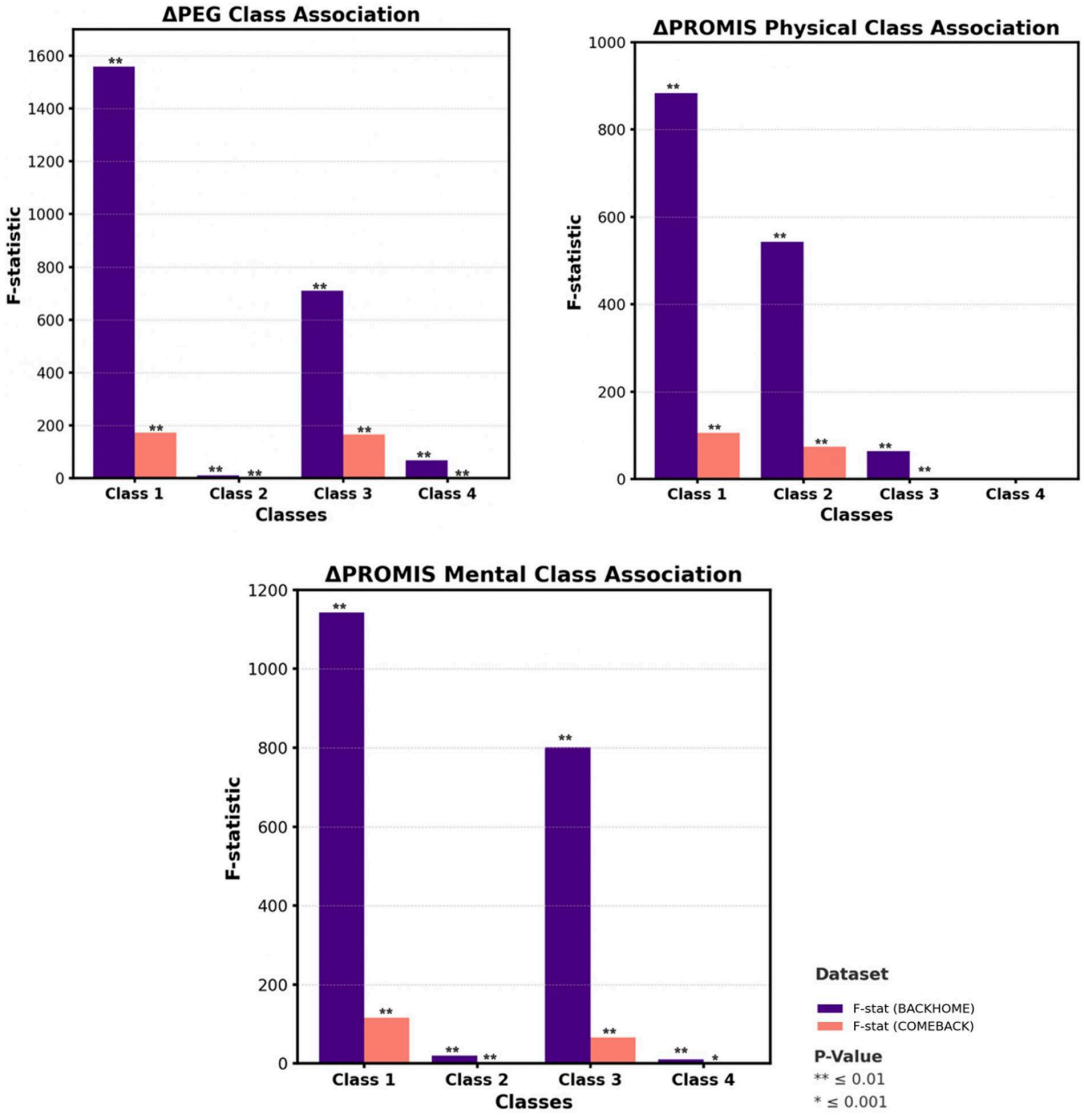
Association of latent classes with changes in clinical outcomes.

We examined changes (Δ) in PEG and PROMIS scores from baseline to follow-up to evaluate how latent class membership was associated with clinical outcomes. Clinical Outcomes Changes were calculated using the following formula: Δ = (Followup - Baseline) /Baseline. As shown in Figure 3, significant class-level differences were observed in ΔPEG, ΔPROMIS Physical, and ΔPROMIS Mental scores. Class 1 showed the highest F-statistics across all outcomes in the train and test datasets (*P* ≤ .001), indicating pronounced changes in pain severity and health-related quality of life over time. Class 3 also demonstrated substantial change, especially in mental health domains. In contrast, Classes 2 and 4 demonstrated smaller F-statistics, indicating more modest clinical changes. These results reinforce the utility of biobehavioral phenotyping through LPA to predict clinical outcomes and inform targeted interventions.

## Discussion

In this study we used LPA to identify biobehavioral phenotypes of cLBP that capture substantial variability in psychological, emotional, and physiological profiles. The heterogeneity within the cLBP population is finding 4 latent classes with unique characteristics related to pain self-efficacy, emotional regulation, and avoidance behaviors. For instance, Class 1, indicating high pain and maladaptive coping behaviors, may benefit from specifically focusing on catastrophizing and fear avoidance and may require more intensive cognitive-behavioral or emotional regulation strategies. In contrast, Class 2, characterized by low pain catastrophizing, may require less intensive bio-behavioral interventions. The replication of the 4-class structure across on the COMEBACK (test) and BACKHOME (train) data sets demonstrates the LPA model’s generalizability and resilience. This consistency shows promise for the model’s application to different cLBP populations, indicating that LPA may successfully stratify patients based on pain-related behavioral and psychological characteristics.

In this work, we identified distinct biobehavioral classes of patients that were generalizable to an external data set. Class 1 was characterized by the poorest pain outcome and maladaptive coping behaviors. Members of this class were noted to have a higher likelihood of exhibiting pain and avoidance behaviors. This group might benefit from therapies that specifically focuses on maladaptive pain behavior, such as catastrophizing and fear-avoidance beliefs. Intensive, multidisciplinary interventions such as exposure-based therapies, cognitive-behavioral therapy (CBT), and emotional regulation techniques, have been shown to successfully improve psychological functioning and lowering pain-related disability.^30–32^ While these interventions are widely supported, individual variability in treatment response suggests that further tailoring of care may be warranted even within this high-risk group. In contrast to Class 1, Class 2 had a more resilient phenotype; in the literature on chronic pain, these individuals are sometimes called “adaptive copers”.^30^^,^^33–35^ These individuals demonstrated little pain interference, high pain self-efficacy, and little emotional distress traits linked to favorable outcomes and effective coping; emotional awareness, self-regulation, and access to strong social support are potential contributors to this resilience. Class 2 patients may be well-treated with enhanced self-care and physical exercise.

Class 3 and Class 4 demonstrated intermediate profiles on various scales, reflecting a range of psychological and behavioral patterns. The specific distinctions between these 2 classes included differences in variables like emotional awareness, pain self-efficacy, and anxiety/depression levels, but they generally represented patients with moderate scores on coping and avoidance measures. For patients in Class 3 we may suggest targeted behavioral coaching to support emotional regulation and adaptive coping. Class 4 indicates that lower-intensity therapies, such as brief cognitive-behavioral or mindfulness-based interventions, may be effective.^36^

This study has several strengths. First, it demonstrates the application of LPA to categorize patients with cLBP based on biobehavioral phenotypes. It offers a deeper insight into patient subgroups and highlights the diversity within this population. In contrast, Newman et al.^37^ distinguished classes based on demographic and socioeconomic characteristics. Wettstein et al.^38^ identified a “low well-being” cluster characterized by the highest pain intensity, the greatest pain-related disability (pain interference), and the lowest levels of psychosocial resources, including reduced mental health, lower resilience, and limited social support. Rabey et al.^39^ derived clusters using a broad range of psychological indicator variables. Like our findings, Obbarius et al.^21^ created subgroups based on emotional distress and pain burden but lacked the focus on coping mechanisms observed in our classes. The findings are strengthened using baseline data from two datasets: The large BACKHOME and COMEBACK and our LPA model showed significant differences in clinical outcomes (Figure 3), indicating clinical relevance and utility in patient-specific outcome prediction. The model’s generalization across both datasets enhances its reliability and suggests that LPA can be a valuable tool for investigating complex, multifactorial conditions like cLBP.

However, this study also has limitations. One of these limitations is the LPA methodology; a common problem with LPA is that results invite pre-mature interpretation despite the need for further cross-study and longitudinal assessment.^40^^,^^41^ It depends on the researcher’s conclusion and clinical expert opinion to determine whether the latent classes convey meaningful entities, which introduces a degree of interpretive bias into the analysis. Another limitation concerns the balance between parsimony and complexity in class selection. The four-class model was selected to preserve interpretability and simplicity. However, this strategy may have missed more complex subgroups within the cLBP population. Although more intricate models might yield more in-depth information, they run the risk of overfitting, which could reduce their usefulness in clinical settings. We also considered the Lo-Mendell-Rubin (LMR) likelihood ratio test when evaluating our model. The LMR *P* values for models range from 1 to 10 classes. All *P* values were greater than .05, indicating that the improvement in model fit when adding additional classes was not statistically significant. The LMR test is conservative, sensitive to sample size and class separation, and frequently inadequate in detecting class distinctions in complex or diverse samples.^42^^,^^43^ While our model selection was considered by multiple criteria (AIC, BIC and entropy), Future studies may consider sensitivity analyses using random subsamples further to evaluate the stability of the class structure in large datasets. A further limitation concerns external generalization. Although the model was validated using 2 data sets (BACKHOME and COMEBACK), additional generalization with independent datasets would enhance the robustness of the findings. In addition, both study samples were predominantly non-Hispanic White and highly educated, which may limit the findings’ representativeness and influence the latent classes’ generalizability. Future research should aim to replicate these results in longitudinal datasets, across diverse clinical populations, and across geographic regions to strengthen the model’s generalizability and relevance. We also plan future studies to expand the biopsychosocial domains included by leveraging the rich, deep phenotyping data captured in COMEBACK, including spinal and brain imaging, biomechanical assessments, and biospecimens from saliva, blood and stool. Furthermore, emotional awareness, self-regulation, and social support availability have been suggested as critical resilience variables in light of the resilient profile shown in Class 2. The psychological qualities of this subgroup may aid in the design of future studies on protective psychological mechanisms and the development of preventative or resilience-enhancing therapies that may assist more vulnerable cLBP patients.

The findings from this study are in line with earlier longitudinal studies that attempted to classify a patient’s risk for persistent cLBP^10^^,^^44^^,^^45^ and indicates the potential for LPA to inform more personalized treatment approaches for cLBP, tailoring interventions to the specific biobehavioral profiles of patient subgroups. Future research should explore the integration of biobehavioral phenotypes with structural, mechanical, genetic, and movement patterns in longitudinal datasets and explore the efficacy of targeted therapies on these distinct classes to potentially enhance treatment efficacy and patient outcomes.

## Conclusion

In conclusion, our work identifies four distinct latent classes in patients with cLBP, characterized by variations in key psychosocial variables such as emotional awareness, self-regulation, anxiety, and depression. These findings provide a valuable understanding of the behavioral phenotypes of cLBP, which may inform more effective and personalized treatment strategies. By distinguishing these subgroups, this work contributes a deeper insight into the heterogeneity within the cLBP population, offering a pathway for enhanced clinical interventions.

## Supplementary Material

pnaf095_Supplementary_Data

## Data Availability

The data that support the findings of this study are openly available in the Vivli repository at https://doi.org/10.25934/PR00010820 and https://doi.org/10.25934/PR00010819.
