# Supplementary material for: Biobehavioral phenotypes of chronic low back pain: Psychosocial subgroup identification using latent profile analysis
Source: Pain Med. 2025 Jul 25;27(1):15–25. doi: 10.1093/pm/pnaf095 (PMC12419233; doi:10.1093/pm/pnaf095)
Supplement: pnaf095_Supplementary_Data [file pnaf095_supplementary_data.docx]

**Appendices**

**A: Data Dictionary**

Below, we will provide a more detailed explanation of the variables used in this study.

**PEG:** The PEG Score is calculated from the mean of the following three questions each range 0-10:

- What number best describes your pain on average, in the past week?
- What number best describes how, during the past week, pain has interfered with your enjoyment of life?
- What number best describes how, during the past week, pain has interfered with your general activity?

(0 = no pain; 10 = worst pain imaginable)

**PROMIS Pain Interference:**  Score is calculated as the sum of the following 4 values each ranged 1-5: In the past 7 days, how much did pain interfere with your:

- day to day activities?
- work around the home?
- ability to participate in social activities?
- household chores?

Where 1 corresponds to ‘not at all’; 2 corresponds to ‘a little bit‘; 3 corresponds to ‘somewhat‘;4 corresponds to ‘quite a bit‘ and 5 corresponds to ‘very much‘.

**Fear Avoidance:** This is the FABQ-P (Fear Avoidance Beliefs with Physical Activity) score, which is calculated as 4 times the mean of their answers to the following questions:

- Physical activity makes my pain worse
- Physical activity might harm my back
- I should not do physical activities which (might) make my pain worse
- I cannot do physical activities which might make my pain worse

Where responses are on a scale from 0-6 where 0 means completely disagree and 6 is completely agree, thus a higher score means more fear.

**Duration Low Back Pain**: duration of low back pain (total years).

**Average Anxiety and Depression**: T-scores from PROMIS Anxiety and Depression scales.

- PROMIS Depression: (range:41.0-79.4, higher=more depressed)
- PROMIS Anxiety : (range 40.3-83.1; higher=more severe anxiety)

**Not Distracting**: This is the MAIA-SF (Multi-dimensional Assessment of Interoceptive Awareness, v2) – Not- Distracting Sub-Score. Participants are asked to answer the following question, Please indicate how often each statement applies to you generally in daily life:

- I ignore physical tension or discomfort until they become more severe.
- I distract myself from sensations of discomfort.
- When I feel pain or discomfort, I try to power through it.
- I try to ignore pain.
- I push feelings of discomfort away by focusing on something.
- When I feel unpleasant body sensations, I occupy myself with something else, so I don’t have to feel them.

Where responses are on a scale from 0-5 where 0 means never and 5 means always.

The non-distracting sub-score is then calculated as the mean of 5 minus each of their responses, thus a higher score means less distracting.

**Emotion Aware:** This is the MAIA-SF Emotional Awareness Sub-Scale score which is the mean of their answers to the following questions:

• I notice how my body changes when I am angry.

• When something is wrong in my life I can feel it in my body.

• I notice that my body feels different after a peaceful experience.

• I notice that my breathing becomes free and easy when I feel comfortable.

• I notice how my body changes when I feel happy / joyful.

where responses are on a scale from 0-5 where 0 means never and 5 means always, thus a higher score means more awareness.

**Self Regulation:** This is the MAIA-SF Self-Regulation Sub-Scale which is the mean of their answers to the following questions:

• When I feel overwhelmed, I can find a calm place inside.

• When I bring awareness to my body I feel a sense of calm.

• I can use my breath to reduce tension.

• When I am caught up in thoughts, I can calm my mind by focusing on my body/breathing.

where responses are on a scale from 0-5 where 0 means never and 5 means always, thus a higher score means more self-regulation.

**Pain Catastrophizing**: This is the PCS-6 score, which is calculated as 3 times the mean of helplessness sub-score, magnification sub-score, and rumination sub-score.

The helplessness sub-score is calculated as the mean of their answers to the following questions:

- It’s awful and I feel that it overwhelms me
- I feel I can’t stand it anymore

The magnification sub-score is calculated as the mean of their answers to the following questions:

- I become afraid that the pain will get worse
- I keep thinking about how much it hurts

The rumination sub-score is calculated as the mean of their answers to the following questions:

- I keep thinking about how badly I want the pain to stop
- I wonder whether something serious may happen

For all questions, responses are on a scale of 0-4 where:

- 0: Not at all
- 1: To a slight degree
- 2: To a moderate degree
- 3: To a great degree
- 4: All the time

Thus, a higher score means more catastrophizing.

**Self-Efficacy:** This is the PSEQ-4 (Pain Self-Efficacy) score, which is calculated as 4 times the mean of their answers to the following questions:

- I can cope with my pain in most situations.
- I can still do many of the things I enjoy doing, such as hobbies or leisure activity, despite pain
- I can still accomplish most of my goals in life, despite the pain.
- I can live a normal lifestyle, despite the pain.

Where responses were originally on a scale of 1-6 with 1 meaning not at all confident and 6 meaning completely confident. These responses were then mapped to the following values for calculation:

1: 0

2: 1.2

3: 2.4

4: 3.6

5: 4.8

6: 6

**B  : One-way ANOVA test with Bonferroni Adjusted P-value for the (A) Train set and (B) Test set.**

**A:**

| ***P* value of Bonferroni(Train set)** | | | | | |
| --- | --- | --- | --- | --- | --- |
| **Variables** |  | **Class 1** | **Class 2** | **Class 3** | **Class 4** |
| PEG | | 0.00 | 0.00 | 0.00 | 0.00 |
| PCS6 | | 0.00 | 0.00 | 0.00 | 0.00 |
| Pain self-Efficacy | | 0.00 | 0.00 | 0.00 | 0.00 |
| Fear avoidance | | 0.00 | 0.00 | 0.00 | 0.01 |
| Average Anxiety and Depression | | 0.00 | 0.00 | 0.00 | 0.00 |
| Duration Low back pain | | 0.01 | 0.00 | 0.00 | 0.00 |
| MAIA2: Emotional awareness | | 0.00 | 0.00 | 0.01 | 0.00 |
| MAIA2:Not distracting | | 0.00 | 0.00 | 0.01 | 0.01 |
| MAIA2:self-Regulation | | 0.00 | 0.00 | 0.01 | 0.01 |

**B:**

| ***P* value of Bonferroni ( Test set )** | | | | | |
| --- | --- | --- | --- | --- | --- |
| **Variables** |  | **Class 1** | **Class 2** | **Class 3** | **Class 4** |
| PEG | | 0.00 | 0.00 | 0.00 | 0.01 |
| PCS6 | | 0.00 | 0.01 | 0.00 | 0.00 |
| Pain self-Efficacy | | 0.00 | 0.00 | 0.01 | 0.00 |
| Fear avoidance | | 0.00 | 0.00 | 0.00 | 0.01 |
| Average Anxiety and Depression | | 0.00 | 0.00 | 0.01 | 0.00 |
| Duration Low back pain | | 0.01 | 0.01 | 0.00 | 0.00 |
| MAIA2: Emotional awareness | | 0.01 | 0.01 | 0.01 | 0.00 |
| MAIA2:Not distracting | | 0.01 | 0.01 | 0.01 | 0.01 |
| MAIA2:self-Regulation | | 0.00 | 0.01 | 0.01 | 0.01 |

**C:**  **Z scores for the (A) Train set and (B) Test set**

| ***Z scores* (Train set)** | | | | | |
| --- | --- | --- | --- | --- | --- |
| **Variables** |  | **Class 1** | **Class 2** | **Class 3** | **Class 4** |
| PEG | | -0.46 | 0.24 | -0.04 | -0.08 |
| PCS6 | | -0.47 | 0.24 | -0.03 | -0.03 |
| Pain self-Efficacy | | 0.46 | -0.24 | 0.04 | 0.06 |
| Fear avoidance | | -0.39 | 0.19 | 0.00 | -0.05 |
| Average Anxiety and Depression | | -0.45 | 0.24 | -0.10 | -0.09 |
| Duration Low back pain | | -0.15 | 0.03 | -0.04 | -0.01 |
| MAIA2: Emotional awareness | | -0.15 | 0.08 | -0.04 | -0.01 |
| MAIA2:Not distracting | | 0.08 | -0.04 | -0.00 | 0.02 |
| MAIA2:self-Regulation | | 0.08 | -0.04 | 0.01 | 0.03 |

**A:**

| **Z scores ( Test set)** | | | | | |  |
| --- | --- | --- | --- | --- | --- | --- |
| **Variables** |  | **Class 1** | **Class 2** | **Class 3** | **Class 4** |  |
| PEG | | 0.19 | 0.24 | -0.01 | -0.21 |  |
| PCS6 | | 0.17 | 0.20 | 0.04 | -0.23 |  |
| Pain self-Efficacy | | -0.17 | -0.09 | -0.12 | 0.29 |  |
| Fear avoidance | | 0.12 | 0.13 | 0.06 | -0.22 |  |
| Average Anxiety and Depression | | -0.47 | 0.08 | 0.06 | -0.22 |  |
| Duration Low back pain | | | -0.04 | 0.3 | -0.19 | 0.13 |
| MAIA2: Emotional awareness | | 0.14 | 0.08 | -0.06 | 0.03 |  |
| MAIA2:Not distracting | | 0.02 | -0.05 | -0.00 | 0.04 |  |
| MAIA2:self-Regulation | | 0.06 | 0.1 | -0.15 | 0.18 |  |

**B:**
